# Supplementary material for: Mediterranean Diet Effect on the Intestinal Microbiota, Symptoms, and Markers in Patients with Functional Gastrointestinal Disorders
Source: Microorganisms. 2024 Sep 28;12(10):1969. doi: 10.3390/microorganisms12101969 (PMC11509143; doi:10.3390/microorganisms12101969)
Supplement: Supplementary file 1 [file microorganisms-12-01969-s001.zip › microorganisms-3189690-supplementary.pdf]

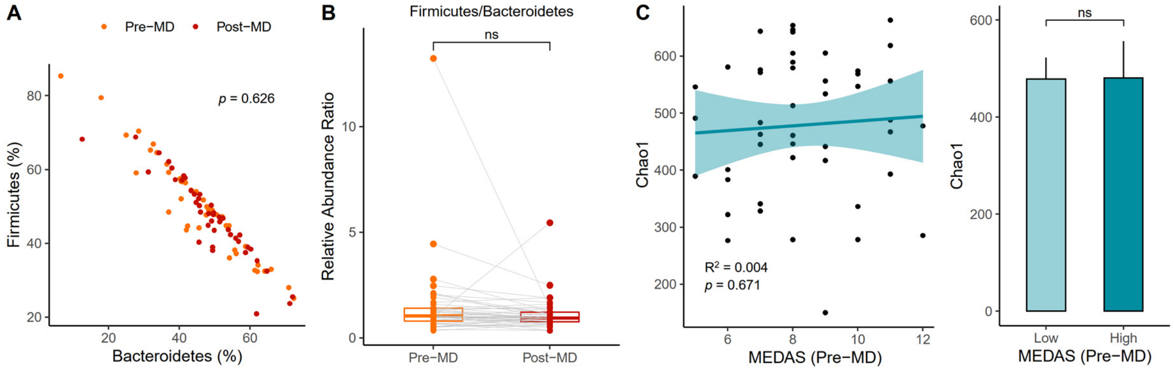

Figure S1. Characteristics of bacteria in the gut of pre- and post-MD participants. (A) Relationship between Firmicutes and Bacteroidetes. (B) Firmicutes/Bacteroidetes ratio (C) Chao1 richness according to adherence to the MD based on the pre-MD MEDAS score (left) and establishing low (≤ 9) and high (> 9) as categories (right). MD, Mediterranean Diet; ns, not significant; error bars represent 95% CI

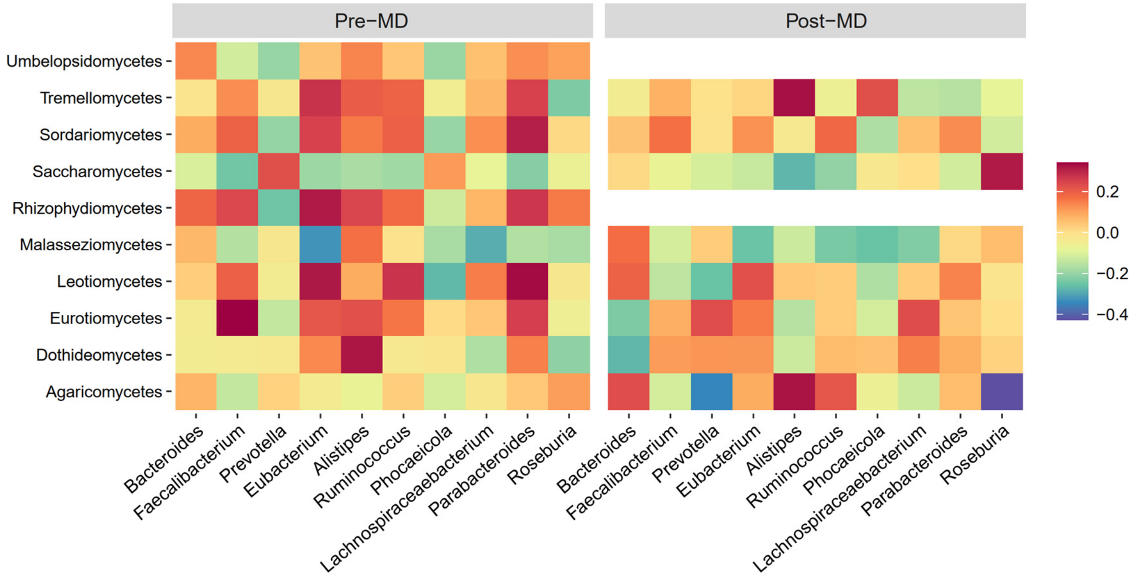

Figure S2: Relationship between fungi and bacteria pre- and post-MD. Correlations with Spearman's test between fungal classes and bacterial genera pre- and post-MD.

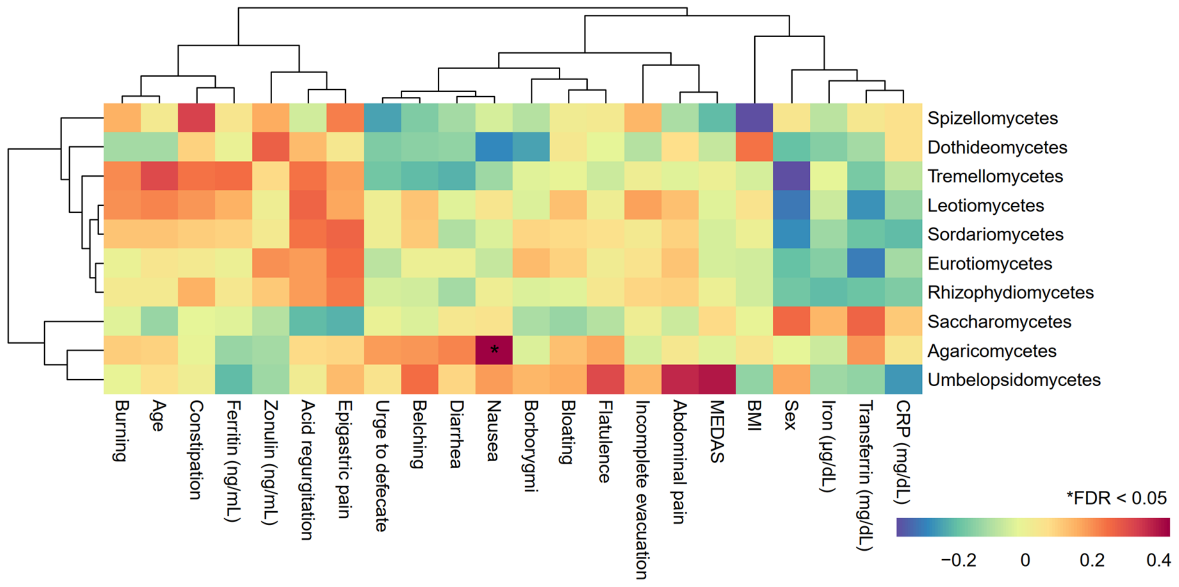

Figure S3. Associations between GI disorders, biomarkers, sample characteristics, and the top 10 pre-MD fungal classes. Hierarchical grouping of correlations with Spearman's test between study variables and pre-MD fungal classes. GI disorders were evaluated with ordinal response variables (0 = Absence; 1 = Mild; 2 = Moderate; 3 = Severe). Sex was coded as numerical variable (0 = Man and 1 = Woman) denoting positive associations with women and negative associations with men. CRP, C-Reactive Protein; FDR, False Discovery Rate.
